# Comparative Evaluation of a Medical Large Language Model in Answering Real-World Radiation Oncology Questions: Multicenter Observational Study

**DOI:** 10.2196/69752

**Published:** 2025-09-23

**Authors:** Fabio Dennstädt, Max Schmerder, Elena Riggenbach, Lucas Mose, Katarina Bryjova, Nicolas Bachmann, Paul-Henry Mackeprang, Maiwand Ahmadsei, Dubravko Sinovcic, Paul Windisch, Daniel Zwahlen, Susanne Rogers, Oliver Riesterer, Martin Maffei, Eleni Gkika, Hathal Haddad, Jan Peeken, Paul Martin Putora, Markus Glatzer, Florian Putz, Daniel Hoefler, Sebastian M Christ, Irina Filchenko, Janna Hastings, Roberto Gaio, Lawrence Chiang, Daniel M Aebersold, Nikola Cihoric

**Affiliations:** 1 Inselspital, Department of Radiation Oncology Bern University Hospital University of Bern Bern Switzerland; 2 Department of Radiooncology and Radiotherapy University Hospital Heidelberg Heidelberg Germany; 3 Department of Radiation Oncology Cantonal Hospital Winterthur Winterthur Switzerland; 4 Radiation Oncology Center Mittelland Cantonal Hospital Aarau Aarau Switzerland; 5 Department of Radiation Oncology Hospital of Bolzano Teaching Hospital of Paracelsus Medical University Bolzano Italy; 6 Department of Radiation Oncology University Hospital Bonn University of Bonn Bonn Germany; 7 Department of Radiation Oncology University Hospital Tübingen Tübingen Germany; 8 Department of Radiation Oncology Klinikum rechts der Isar Technical University of Munich Munich Germany; 9 Institute of Radiation Medicine Department of Radiation Sciences Helmholtz Zentrum Munich Germany; 10 German Cancer Consortium Partner Site Munich Munich Germany; 11 Department of Radiation Oncology Health Ostschweiz (HOCH), Cantonal Hospital St. Gallen St. Gallen Switzerland; 12 Department of Radiation Oncology University Hospital Erlangen Friedrich-Alexander University Erlangen-Nürnberg Erlangen Germany; 13 Department of Radiation Oncology University Hospital of Zurich Lausanne Switzerland; 14 Department of Radiation Oncology University Hospital of Lausanne Lausanne Switzerland; 15 Inselspital, Department of Neurology Bern University Hospital University of Bern Bern Switzerland; 16 School of Medicine University of St. Gallen St. Gallen Switzerland; 17 Faculty of Medicine Institute for Implementation Science in Health Care University of Zurich Zurich Switzerland; 18 Swiss Institute of Bioinformatics Lausanne Switzerland

**Keywords:** large language models, natural language processing, artificial intelligence, radiation oncology, Llama-3, benchmarking, evaluation

## Abstract

**Background:**

Large language models (LLMs) hold promise for supporting clinical tasks, particularly in data-driven and technical disciplines such as radiation oncology. While prior evaluation studies have focused on examination-style settings for evaluating LLMs, their performance in real-life clinical scenarios remains unclear. In the future, LLMs might be used as general AI assistants to answer questions arising in clinical practice. It is unclear how well a modern LLM, locally executed within the infrastructure of a hospital, would answer such questions compared with clinical experts.

**Objective:**

This study aimed to assess the performance of a locally deployed, state-of-the-art medical LLM in answering real-world clinical questions in radiation oncology compared with clinical experts. The aim was to evaluate the overall quality of answers, as well as the potential harmfulness of the answers if used for clinical decision-making.

**Methods:**

Physicians from 10 departments of European hospitals collected questions arising in the clinical practice of radiation oncology. Fifty of these questions were answered by 3 senior radiation oncology experts with at least 10 years of work experience, as well as the LLM Llama3-OpenBioLLM-70B (Ankit Pal and Malaikannan Sankarasubbu). In a blinded review, physicians rated the overall answer quality on a 5-point Likert scale (quality), assessed whether an answer might be potentially harmful if used for clinical decision-making (harmfulness), and determined if responses were from an expert or the LLM (recognizability). Comparisons between clinical experts and LLMs were then made for quality, harmfulness, and recognizability.

**Results:**

There were no significant differences between the quality of the answers between LLM and clinical experts (mean scores of 3.38 vs 3.63; median 4.00, IQR 3.00-4.00 vs median 3.67, IQR 3.33-4.00; *P*=.26; Wilcoxon signed rank test). The answers were deemed potentially harmful in 13% of cases for the clinical experts compared with 16% of cases for the LLM (*P*=.63; Fisher exact test). Physicians correctly identified whether an answer was given by a clinical expert or an LLM in 78% and 72% of cases, respectively.

**Conclusions:**

A state-of-the-art medical LLM can answer real-life questions from the clinical practice of radiation oncology similarly well as clinical experts regarding overall quality and potential harmfulness. Such LLMs can already be deployed within the local hospital environment at an affordable cost. While LLMs may not yet be ready for clinical implementation as general AI assistants, the technology continues to improve at a rapid pace. Evaluation studies based on real-life situations are important to better understand the weaknesses and limitations of LLMs in clinical practice. Such studies are also crucial to define when the technology is ready for clinical implementation. Furthermore, education for health care professionals on generative AI is needed to ensure responsible clinical implementation of this transforming technology.

## Introduction

Large language models (LLMs) are a form of generative artificial intelligence (AI). They have shown promising capabilities in answering questions from various medical and nonmedical domains [1]. For example, the LLM Med-PaLM 2 developed by Google correctly answered 86.5% of medical questions in the style of the United States Medical Licensing Examination (USMLE) [2]. These systems demonstrated success in numerous applications such as medical writing, education, or diagnosis, and are expected to transform the clinical environment [3].

Given that LLMs can integrate extensive domain-specific knowledge, their use as general assistant systems or agents for answering clinical questions is frequently discussed [4]. The LLM would thus give medical advice and be involved in the clinical decision-making process. Early evaluation studies were performed following the substantial performance improvements in LLMs at the end of 2022. These studies have shown the remarkable ability of systems such as ChatGPT (OpenAI, Inc), the Llama (Meta Platforms, Inc) models, or PALM (Google LLC) in answering medical questions [5]. This includes the field of radiation oncology, a highly specialized and technical discipline grounded in computerized information technology, where the application of generative AI therefore holds great potential [6-8].

Most of these evaluation studies have been performed on examination-style questions with predesigned questions in a test setting [6,9,10]. Such evaluation studies (many with single- or multiple-choice questions) allow clear identification of correct and incorrect answers by an LLM. Overall, the performance of LLMs is rapidly improving according to various medical benchmarks. For example, models such as MedPALM-2 have been reported to answer questions “at the level of an expert doctor” [2].

However, a limitation of these studies is that predesigned questions do not accurately reflect real-life clinical situations. Medical questions arising from clinical practice rarely have only one correct “textbook” answer, since they are often open-ended with limited supporting evidence. Therefore, results from currently published evaluation studies do not reflect the performance of LLMs in clinical practice.

At the same time, the performance of LLMs against these benchmarks is rapidly increasing. On one hand, LLMs are becoming larger and more powerful (eg, GPT-3.5 incorporates 175 billion parameters, compared with >1.5 trillion in GPT-4) [11], whereas on the other hand, smaller, optimized, and more efficient models are being developed [12]. These smaller models require less computational power and can operate locally within a clinical environment, eliminating the need for external servers (eg, those used by ChatGPT, Claude [Anthropic PBC], or Gemini [Google LLC]) [12].

We aimed to investigate the performance of Llama3-OpenBioLLM-70B [13], a modern state-of-the-art open medical LLM that can be securely run in a local environment, in answering real-life clinical questions. In a collaborative project between ISROI (International Society for Radiation Oncology Informatics) and DEGRO (German Society for Radiation Oncology), answers given by the LLM were evaluated. Furthermore, results were compared with answers given by clinical experts in a multicentric observational evaluation study.

## Methods

### Study Design

**Figure 1:**
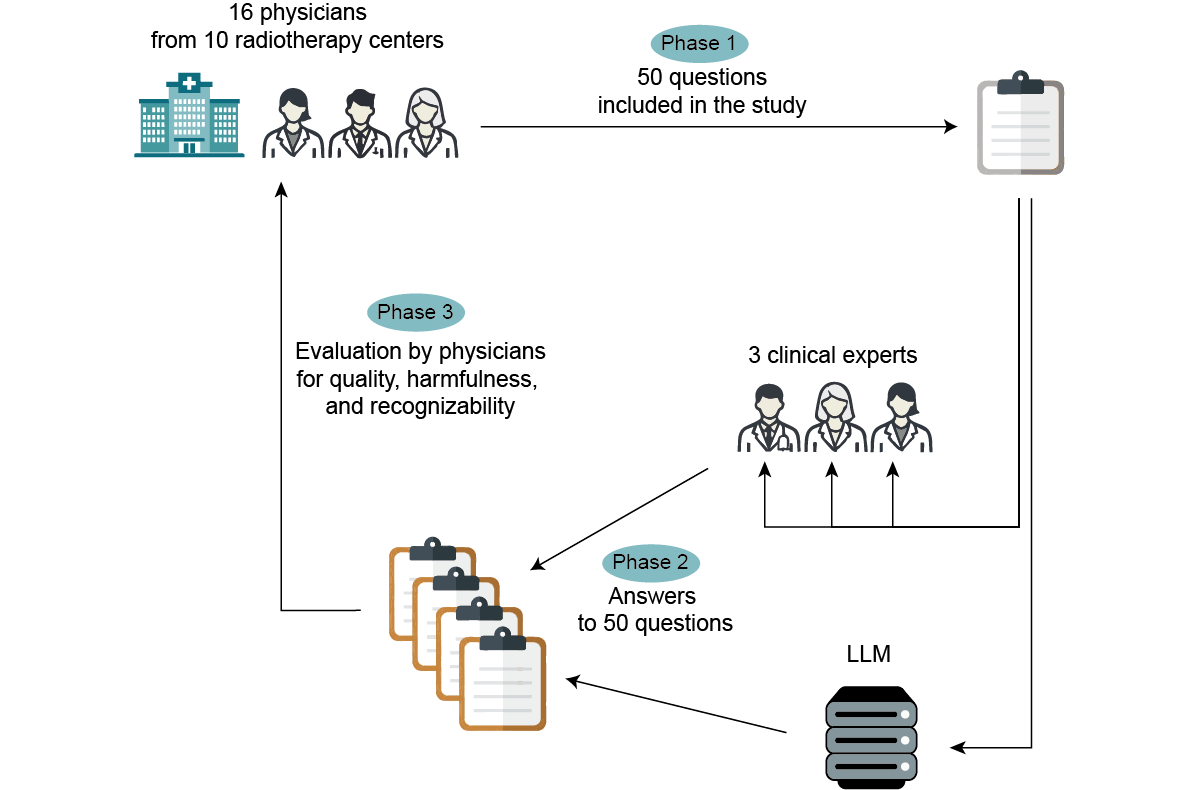
Schematic illustration of the study design. LLM: large language model.

The study had 3 phases (Figure 1). In phase 1, participating radiation oncologists (=physicians) collected questions from radiation oncology clinical practice. In phase 2, clinical experts and a medical LLM answered these questions. In phase 3, participating physicians from phase 1 evaluated the answers given by the experts and the LLM in a blinded review.

The open-source internet-based platform Smart Oncology (Wemedoo AG) [14,15] was used for the collection of questions, the evaluation of answers by participating physicians, and the response submissions by clinical experts.

#### Phase 1: Collection of Questions From Clinical Practice

Participating physicians were recruited among the members of the ISROI as well as from the Digitalization and Artificial Intelligence Focus Group of the DEGRO. Questions were collected over 8 weeks, from the May 22 to June 16, 2024, by 20 participating radiation oncologists from 10 radiation oncology departments in European hospitals. These included the radiotherapy departments of the University Hospital of Bern, the Cantonal Hospital of Winterthur, the Cantonal Hospital of Aarau, the Cantonal Hospital of St. Gallen, the University Hospital of Zurich, the University Hospital of Lausanne, the State Hospital of Bolzano, the University Hospital of Tübingen, the Technical University of Munich, and the University Hospital of Erlangen. Participating physicians included both residents and senior physicians.

The physicians were instructed to document questions that arose during their daily clinical practice as radiation oncologists as follows:

Generative AI is transforming medicine, and, in the future, clinicians may consult an AI agent when faced with a medical question that arises during clinical work. Please write down any questions that you would ask such an AI agent if it were already available in your clinic.

Due to ethical and data privacy concerns, clinicians were instructed not to record any questions that included patients’ personal information. While the idea was to collect questions from the clinical routine of radiotherapy, valid questions included those that were not primarily related to radiotherapy (eg, a valid but not primarily radiotherapeutic question might be “What is the maximum dose of paracetamol I can give a patient with side effects during treatment?”).

Furthermore, for language consistency, clinicians were instructed to record the questions in English.

Of the collected questions, 50 were randomly selected for the study using a pseudorandom number generation algorithm implemented in Python (Python Software Foundation). After screening the initial questions, the study coordinators assigned the questions to one of the following thematic categories: “prostate,” “head and neck,” “gynecological” (including breast cancer), “genitourinary” (excluding prostate cancer), “central nervous system,” “lung,” “palliative,” and “other.”

#### Phase 2: Answering the Questions

Three radiation oncologists from different centers of the community of ISROI and DEGRO with profound knowledge in radiation oncology were selected as clinical experts to answer the questions. The clinical experts had at least 5 years of clinical experience post specialization and, therefore, at least 10 years of work experience in radiation oncology. The clinical experts were given the following instructions:

Please answer the given question. Imagine this question being asked of you by a colleague in a dialogue or via mail. It is up to you how detailed you want to answer this question. The aim is to provide a helpful and qualitatively valuable answer.

The clinical experts were allowed to consult medical literature or conduct internet research as needed to look up details while answering the questions. To avoid bias, the clinical experts were not allowed to use any form of generative AI (eg, such as ChatGPT). For each question, the clinical experts indicated the difficulty of the question on a 5-point Likert scale (1: very easy, 2: easy, 3: intermediate, 4: difficult, and 5: very difficult). Based on the difficulty score, a question was classified as easy (score <2.66), intermediate (score 2.66-3), or difficult (score >3).

The same questions were also answered by the medically fine-tuned Llama3 LLM OpenBioLLM-70B [13]. The LLM was selected for the study as one of the best-performing open-source LLMs across several medical question-answering benchmarks such as MedMCQA, MMLU Medicine, and PubMedQA, while being an open-source model that can be run on a local system [13].

To locally execute the model, a quantized 5-bit GGUF version [16] was used using llama.cpp (Georgi Gerganov and community) [17]. The model was run on a Mac Studio with an Apple M2 Max using a simple Python script (available on GitHub [18]). A standardized prompt was used for all 50 questions. This prompt was manually created by the study coordinators in an unsystematic way, without sophisticated prompting techniques. The purpose of the prompt was to instruct the model to provide factually correct, helpful, and concise answers. The prompt followed the Llama3 formatting as published by Meta AI and included special tokens [19]. The used prompt was

“<|begin_of_text|><|start_header_id|>system<|end_header_id|>

You are a radiation oncology specialist. You are asked a question by a colleague. You give a factual, correct, helpful, and concise answer. The answer should be very brief. <|eot_id|><|start_header_id|>user< |end_header_id|>QUESTION<|eot_id|><|start_header_id|> assistant<|end_header_id|>The answer is:”

The output was capped at 400 tokens (≈300 words). The response of the LLM was used without any modifications.

Further technical details are provided in Multimedia Appendix 1.

#### Phase 3: Evaluation

Question-answer sets were prepared for evaluation by randomly shuffling the order of the 3+1 answers using a pseudo-random number generator algorithm implemented in Python. The answers did not include an indication about their source (ie, clinical expert or LLM).

The question-answer sets were returned to the participating physicians for evaluation. Each answer was independently evaluated by the physician who submitted the question (=questionnaire reviewer) and by a second randomly selected independent participating physician who did not send in the question (=second reviewer).

First, the physicians rated the quality of each answer on a 5-point Likert scale (1: very bad, 2: bad, 3: acceptable, 4: good, and 5: very good). Given the potential for differing perspectives due to varying levels of medical knowledge regarding individual circumstances, there may not always be a single clear answer to an open-ended question. Therefore, radiation oncologists were instructed to base their evaluations on widely accepted medical knowledge rather than relying on personal opinions when evaluating the “overall quality” of an answer.

Second, the physicians marked whether they believed an answer could be potentially harmful if used in clinical decision-making.

Third, they indicated whether they thought an answer was given by a human or by an AI.

All 50 questions together with the answers of the LLM, as well as the evaluations, are provided on GitHub [18].

### Data and Statistical Analysis

The analyses were exploratory and performed using R (version 4.4.2; R Core Team). Unless stated otherwise, continuous variables were presented as median and IQR, while categorical variables were presented as count (ie, % of total). There was no missing data.

We compared the quality of the answers (ie, as a continuous characteristic) between the LLM and clinical experts (ie, the quality) as dependent variables using the Wilcoxon signed rank test. Moreover, the quality was described for the individual thematic groups and the 3 difficulty levels. No further analysis was conducted on these subsets due to the small sample size.

To further account for the potential impact of question difficulty on answer quality, we used a mixed-effects linear regression. In this model, the quality was a dependent variable, source (ie, clinical experts vs LLM) and difficulty were fixed effects, and the question was a random effect. As the second step, the answers of the clinical experts were compared individually to those of the LLM, and the false-discovery rate was applied to correct for multiple comparisons.

Similarly, the length of the answers for the clinical experts was compared with those of the LLM as dependent variables using the Wilcoxon signed rank test.

Finally, we compared the categorical characteristics of the answers between the clinical experts and the LLM (ie, the harmfulness and the recognizability of the answers). As a first step, the cumulative value of the characteristics of the answers from the clinical experts was compared with those of the LLM using the Fisher exact test. These values were treated as independent variables to avoid bias while calculating mean recognizability from categorical variables. As the second step, the answers of the clinical experts were compared individually to those of the LLM as dependent variables using the McNemar test, and the false-discovery rate was applied to correct for multiple comparisons.

All statistical tests were 2-sided and conducted at a significance level of 5%.

### Ethical Considerations

The study was deliberately designed such that no patient- or person-related medical or nonmedical data were used. It therefore does not fall under the jurisdiction of the Federal Act on Research involving Human Beings [20], and no approval from an ethics committee was required. A declaration of nonresponsibility was issued by the ethics committee of the Canton of Bern (Reqw-2025-00059). All of the data (questions, answers, and evaluations) used in this work were generated by members of the research group, who voluntarily participated without compensation. The data was locally processed and stored on a protected server at the University Hospital of Bern.

## Results

### Collected Questions and Length of Answers

A total of 133 questions were collected by 16 of the participating physicians. About 4 of the initial 20 physicians did not submit any questions and did not further participate in the study, and 7 questions were deemed invalid by the study coordinators due to clarity or submission of the question in a language other than English.

**Figure 2:**
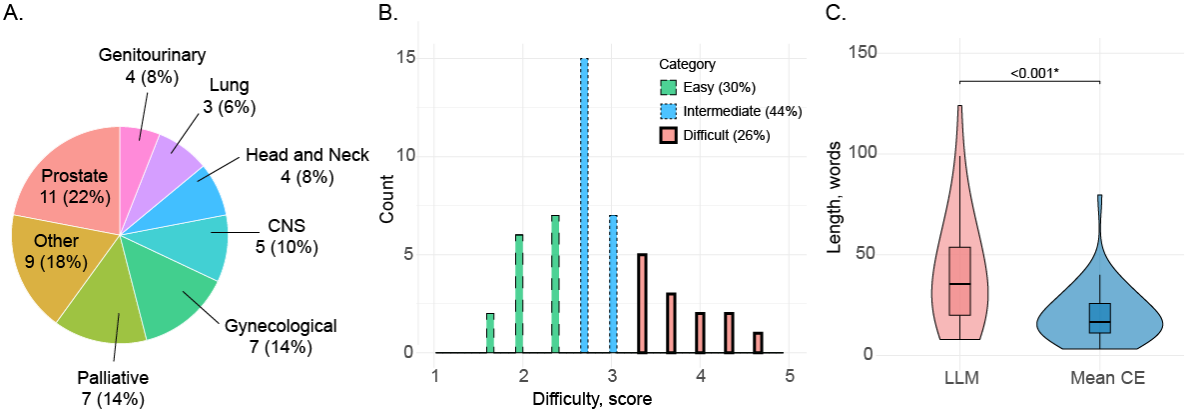
Properties of the collected questions and the length of answers. (A) Thematic distribution of the questions as assigned by the study coordinators, (B) histogram of the difficulty of questions based on the mean difficulty score, (C) box plots with violin plots of the length of answers of the large language model and the mean clinical expert. CE: clinical expert; CNS: central nervous system; LLM: large language model.

The 50 randomly selected questions were mostly categorized as “prostate” (11/50, 22%), “gynecological” (7/50, 14%), “palliative” (7/50, 14%), and “other” (9/50, 18%; Figure 2A).

The difficulty of the questions was 2.67 (IQR 2.33-3.33) points of the 5-point Likert scale. Most questions (22/50, 44%) were of intermediate difficulty, while 26% (13/50) were classified as difficult and 30% (15/50) as easy (Figure 2B).

The length of the questions was 32.0 (IQR 17.25-47.75) words. Clinical experts had significantly shorter answers compared with those generated by the LLM (median 16.67, IQR 11.25-19.96 vs median 35.50, IQR 20.00-40.08 words; *P*<.001; Figure 2C).

### Quality of the Answers

Overall, the LLM answers were deemed to be equally good as or better than the mean clinician answer in 27 cases (54%). In 19 cases (38%), the LLM answer was deemed at least as good as those of the “best clinician.” In 9 cases (18%), the LLM answer was considered worse than those of all 3 clinical experts, and in 2 cases (4%), the LLM answer was considered to be better than those from all 3 clinical experts. 40 of 50 answers (80%) were rated as “acceptable,” “good,” or “very good.”

Regarding the different thematic groups, the quality scores for the LLM were higher than the mean quality of clinical expert answer for “central nervous system” (mean 4.20, SD 0.40 vs mean 3.60, SD 0.88) and “other” (mean 3.78, SD 0.92 vs mean 3.63, SD 1.02) and lower for “head and neck” (mean 3.25, SD 1.30 vs mean 4.00, SD 0.71), “gynecological” (mean 3.29, SD 1.03 vs mean 3.33, SD 0.94), “prostate” (mean 2.82, SD 1.19 vs mean 3.37, SD 1.15), “lung” (mean 3.67, SD 1.25 vs mean 3.89, SD 0.57), “palliative” (mean 3.29, SD 0.70 vs mean 3.86, SD 0.94), and “genitourinary” (mean 3.25, SD 1.30 vs mean 4.00, SD 0.82; refer to Figures S1 and S2 in Multimedia Appendix 2 for assessment of second reviewer). Regarding the difficulty categories, the scores of the LLM compared with the mean clinical expert were 4.00 versus 3.73 for easy, 3.00 versus 3.65 for intermediate, and 3.31 versus 3.49 for difficult questions.

**Figure 3:**
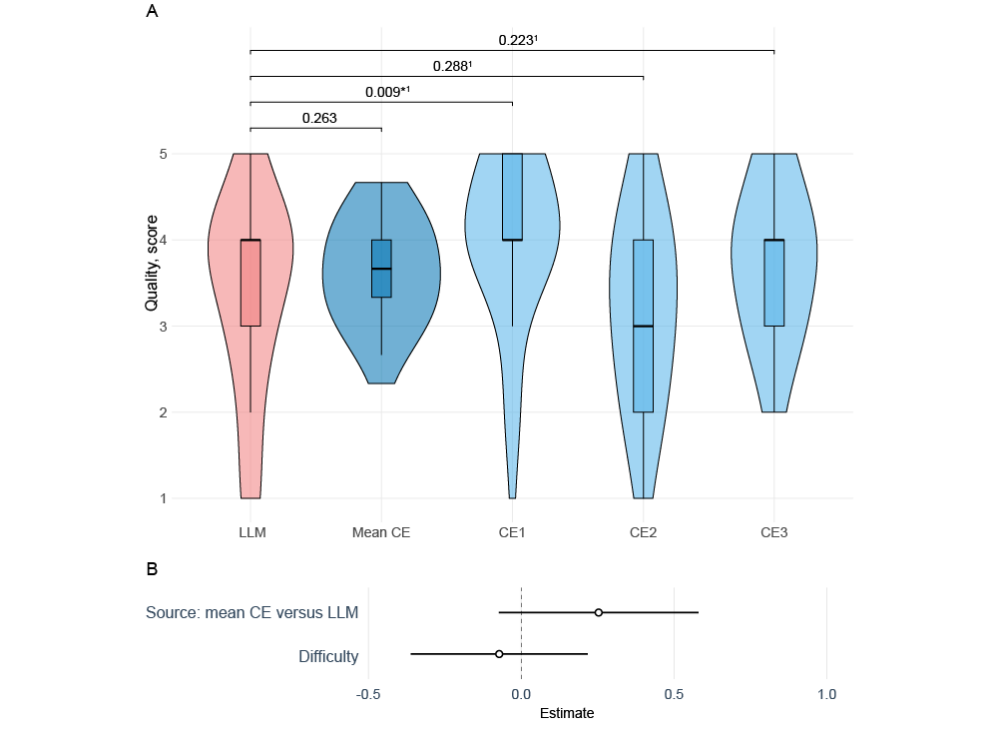
Quality of the answers as assessed by the questionnaire reviewer. (A) Box plots with violin plots for comparison of quality score between the large language model and the mean, as well as individual clinical experts, and (B) association of the quality of answers with their source and difficulty. 1Wilcoxon signed-rank test corrected for multiple comparisons with a false-discovery rate. CE: clinical expert; LLM: large language model.

The quality score of the answers given by the clinical experts had a median of 3.67 (IQR 3.33-4.00; mean 3.63, SD 1.02; range 3.18-4.00), compared to a median of 4.00 (IQR 3.00-4.00; mean 3.38) for the answers given by the LLM, based on the evaluation by the questionnaire reviewer. Whilst there was no statistically significant difference between the LLM and the mean quality of clinical expert answers, the variability between clinical experts was large, with one clinical expert providing answers of significantly higher quality compared with the LLM (Figure 3A). After adjusting for question difficulty as a continuous variable, a positive trend was observed between the quality of the answers from the clinical experts and those of the LLM; however, this association was not statistically significant (Figure 3B).

Similar results were obtained when assessing the quality of the answers according to the second reviewer (Figure S3 in Multimedia Appendix 2).

### Harmfulness of the Answers

**Figure 4:**
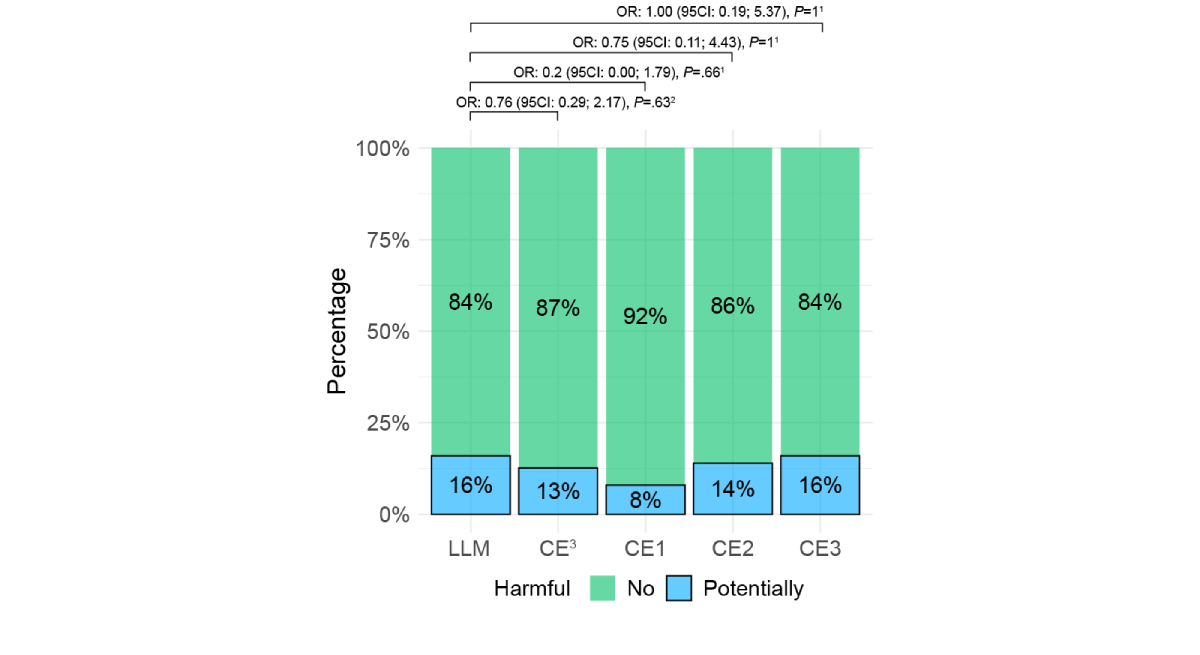
Percentages of answers deemed “potentially harmful” by the questionnaire reviewer. 1McNemar test corrected for multiple comparisons with a false-discovery rate. 2Fisher exact test. 3Cumulative value for clinical experts. CE: clinical expert; LLM: large language model; OR: odds ratio.

According to the questionnaire reviewer, 8/50 (16%) of the answers given by the LLM were considered “harmful” compared with 19/150 (13%) given by the clinical experts (individually 4, 7, and 8 answers). This difference was not statistically significant (Figure 4; Results for the second reviewer are presented in Figure S4 in Multimedia Appendix 2).

### Identification of LLM Versus Clinical Expert

**Figure 5:**
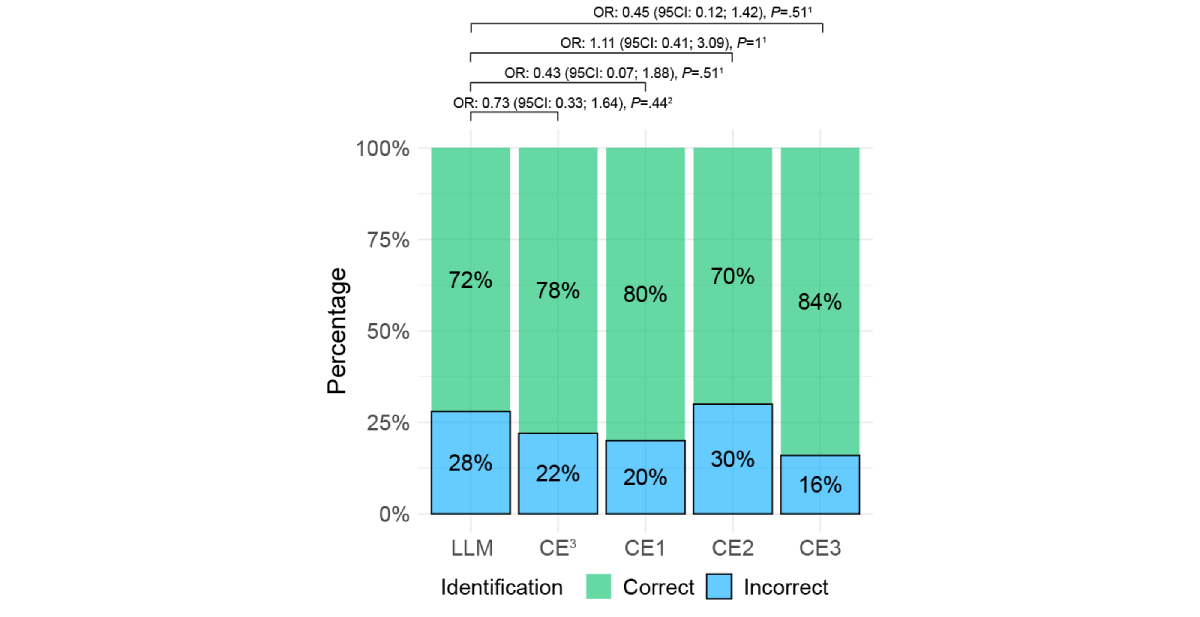
Percentages of correct and incorrect identifications of the source (large language model or clinical expert) by the questionnaire reviewer. 1McNemar test corrected for multiple comparisons with a false-discovery rate. 2Fisher exact test. 3Cumulative value for clinical experts. CE: clinical expert; LLM: large language model; OR: odds ratio.

The physicians correctly identified the source of an answer in most cases, with 78% for answers given by a clinician and 72% for answers given by the LLM (Figure 5; results for the second reviewer are presented in Figure S5 in Multimedia Appendix 2). When the interaction between participant and length was considered, the interaction term between length and clinical experts versus LLM was significant (odds ratio –0.07, 95% CI –0.13 to –0.003; *P*=.04), indicating that the likelihood of correct identification of a clinical expert decrease with the increase in the length of the answers.

## Discussion

### Principal Findings: A State-of-the-Art Medical LLM can Answer Questions From Clinical Practice as Well as Clinical Experts

Our results show that radiation oncologists evaluate the quality of answers given by a modern state-of-the-art medical LLM to questions from clinical practice to be as good as clinical experts with more than 10 years of work experience in radiation therapy. Furthermore, the number of answers considered potentially harmful if used for clinical decision-making was similar between the LLM and clinical experts. The performance of the LLM was high across several topics within radiation oncology.

### Comparison to Prior Work: Evaluation of LLMs in Clinical Practice, Its Challenges, and the Context of This Study

Benchmarking and evaluation studies of LLMs and other forms of generative AI in medicine are of increasing relevance [21]. They are essential to ensure a responsible implementation of these systems in the clinical environment. Regardless of the current uncertainties, LLMs are already frequently used both by clinicians and patients [22]. These systems typically did not undergo a medicine-specific quality assurance process, nor did they receive formal approval as a medical device. The evaluation of LLMs in clinical practice is therefore not just an important but also an urgent task.

At the same time, how to best evaluate the performance of LLMs in general, but particularly for their use in medicine, is a currently unresolved problem [23]. Several approaches have been proposed, including classical examinations, Elo systems [24], or logical benchmarks [1,22].

Our study aimed to mimic a real-world situation whereby clinicians are confronted with a question in daily clinical practice and wish to consult an AI assistant. Furthermore, we compared the performance of the LLM to that of experts in this clinical domain.

We believe that this approach is an essential component of a comprehensive evaluation. First, many questions arising in real life are not examination-style, but open-ended, on topics with only limited data available. Second, the “quality of an answer” needs to be evaluated without a clearly defined ground truth existing. Comparing the answers of an LLM to the gold standard of answers given by clinical experts allows for better interpretation of the results. To our knowledge, our study is the first multicentric evaluation study of an LLM in radiation therapy using questions from real-world clinical practice and comparing the performance of an LLM with that of clinical experts.

Our findings show that the answers given by the medical fine-tuned LLM OpenBioLLM-70B to questions covering various topics in radiation therapy are comparable to those of clinical experts. In a previous study conducted in 2023, we evaluated the performance of ChatGPT (GPT-3.5) in answering radiotherapy questions, with response quality assessed on an analogous 5-point Likert scale by radiation oncologists [6]. In that study, 48% (12 of 25) of open-ended questions were rated as “acceptable,” “good,” or “very good” for helpfulness and safety. While direct comparisons are limited due to different study designs and different datasets, we observed that 80% (40 of 50) of the questions were at least deemed “acceptable” in the current study.

Furthermore, the model seems to perform robustly across different thematic domains. However, it should be noted that the model gave “bad” or “very bad” answers in 10 of 50 cases (20%), and the answers were considered potentially harmful by the questionnaire reviewer in 8 cases (16%).

### Future Directions

#### Are LLMs Ready to Be Used as “AI Assistants” in Clinical Practice?

Determining the threshold at which LLMs are ready for clinical implementation as general AI assistants is challenging [21]. It is interesting that in our study, a similar proportion of responses provided by the average clinical expert was rated as “bad,” “very bad,” or “potentially harmful.” Such results give an idea of how likely it is that the output of an LLM is of poor quality and could hurt clinical decision-making. Studies using real-life tasks and questions are essential to better understand where a threshold for using generative AI in clinical practice can be defined. Based on our results, one may argue that the LLM already performs at an acceptable level and could be used in clinical practice. However, it is important to consider for what clinical application an LLM is to be used [25]. If LLMs are, for example, applied only for medical education purposes of students, of course, one would still want a good system, but one might accept a system that occasionally gives a bad answer. However, in clinical situations where an LLM is involved in the decision-making about the treatment of individual patients with cancer, the AI could cause a lot of harm. For such a purpose, an LLM should not be just as good as some physicians but perform well above that level, effectively minimizing the risk of substantial errors. Unlike an AI system, we would expect a well-trained human physician to know and respect their limitations and also be aware of critical situations when wrong decisions can have severe consequences. Making up incorrect statements and claiming things that are not backed by facts would be considered irresponsible and dangerous behavior by a human. Such behavior is a known issue in LLMs under the term “hallucinations,” which remains an unresolved issue of current generative AI systems [26]. For that reason, we would currently discourage the use of LLMs as general AI assistants in clinical practice.

It remains a valid question when these systems are ready for clinical practice. For our study, we deliberately chose an open-source model that is optimized to the medical domain, instead of general, better-known models provided by private companies (eg, ChatGPT, Claude, or Gemini). A model such as OpenBioLLM-70B can be run in a local environment with all data staying within the hospital and avoiding the transmission of sensitive health care information to an external stakeholder.

From a purely technical perspective regarding setting up and running such a system in a local hospital environment, the technology appears to be ready. The required resources to set up such a system appear manageable, and it operated on consumer-grade hardware designed for household use. Many legal and regulatory issues must be resolved, and multifaceted quality assurance must be done before LLMs can become helpful AI assistants in clinical practice. Given the fast pace of the development of generative AI, we believe that LLMs will soon achieve higher performance compared with most clinical experts in such benchmarking studies. Additional performance gains may be obtained by adding context-specific information in the form of guidelines in full via retrieval augmented generation systems [27]. Beyond that, newer systems will not only process text data but also include multimodal medical data [28,29].

Future studies will therefore need to focus primarily on whether the use of AI systems leads to an improvement of processes, decision-making, and care in the clinical environment.

#### Education of Health Care Professionals for Clinical Application of AI

LLMs and other forms of generative AI will continue to improve, and it is expected that AI will transform health care and clinical practice [29]. Nevertheless, an AI system will never be perfect, and despite the rapid advancements and concerns associated with it, the technology will not replace human doctors in the foreseeable future [30]. AI can help to process huge amounts of data and greatly support health care, but it ultimately remains a tool. Radiation oncology is traditionally a technical and data-driven discipline using high-level technology for treatment delivery. Academic evaluation studies can help to provide more data on both the potential and limitations of the technology. However, the most important measure to mitigate risks and to ensure that clinically applied AI ultimately benefits patients is to focus on the training of health care professionals on the topic [31,32]. Clinicians may not need to have profound knowledge of the architecture or development of AI systems, but need to be educated regarding the limitations and weaknesses of the technology they use in clinical practice. Contemporary modern radiation oncology already necessitates competence in handling computer systems and professional software for various tasks such as case evaluation, radiation treatment planning, and documentation [33,34]. The next generation of radiation oncology, in which LLMs and AI will play an important role, will require a new set of skills and knowledge, including a better understanding of the technology. Medical physicists have traditionally been directly involved in the technical developments of radiation oncology, and it has been proposed that teaching in AI should become an integral part of the professional education [35]. Radiation oncology is an interdisciplinary medical discipline, and professional education in AI will be necessary also for physicians, as well as for radiation therapists [31]. As AI is becoming an integral part of health care, professionals will need to better understand and be taught how the technology works. This will be fundamentally important for the safe integration of AI in clinical practice and will enable clinicians to actively participate in the implementation phase and define their needs and problems in daily clinical life that could be addressed using LLMs and other forms of generative AI.

### Strengths and Limitations

We used a structured and elaborate methodology for our evaluation study, which contributes to its strengths. First, we used questions arising in real-life clinical practice of radiation oncology. In comparison to studies using artificial examination-style questions, better conclusions regarding the application of LLMs in clinical practice can therefore be drawn from the results. Second, we involved 10 radiation therapy centers from different European hospitals to obtain a diverse and representative set of questions and topics relevant to the radiation oncology community. Third, we compared the answers of the LLM to those of clinical experts mimicking a real-life situation in which a physician might ask a colleague (eg, via mail) for advice. Fourth, the answers given by the clinical experts and the LLM were evaluated by peers in a blinded manner to avoid a potential bias.

While our study therefore gives new insights into the evaluation of LLMs in clinical practice, it also has several limitations. First, while the aim was to investigate the performance of LLMs on questions from clinical practice of radiation therapy, no real patient or person-related health care data were used. This is a considerable limitation, as many questions in daily clinical life stem from patient-specific information. Second, assessing the overall “quality of an answer” is challenging, as clinician evaluations are inherently subjective and may vary. This quality cannot be accurately measured using individual intuition or majority consensus and likely comprises dimensions such as safety, helpfulness, and style. Since we did not expect to gain other insights (based on the results from our previous study), and to limit the effort for the study participants, we focused solely on the perceived overall quality of each answer as the primary outcome measure. Third, it is also live whether an answer is perceived as “acceptable” or “potentially harmful.” The results of our study allow some qualitative comparison, since the answers of clinicians and LLM were evaluated alike. However, interpretation of results regarding exact quantitative values is limited. Fourth, due to the considerable effort in conducting the evaluation, we only involved 3 clinical experts, which is a limited number. The questions used in our study consisted of 8 different thematic categories. As radiation oncologists usually specialize in some subspecialty, they do not have the same level of expertise on all topics. In general, the fact that only 3 clinical experts were involved in our study may limit the generalizability of the results. More extensive, but therefore laborious studies will be required in the future to obtain a more general data basis. Finally, evaluators were able to identify which answers were given by LLM and which by a clinical expert in most cases. We hypothesize that this is due to the different language style used by the LLM, generating considerably longer answers. Likely, the identification of whether an answer was given by an LLM or not may have an element of unconscious bias when rating the quality of that answer.

### Conclusions

The answers given by a state-of-the-art medical LLM to real-life clinical questions from radiation oncology practice seemed comparable to those from clinical experts regarding both overall quality and potential harmfulness. Such LLMs can be deployed in a local hospital setting at an affordable cost. However, at the moment, they do not seem to be ready to be used as “general AI assistants” in the clinic. Despite seemingly satisfactory LLM performance, considerable limitations and issues remain. Evaluation studies based on real-life situations are needed to better understand the real-life performance of LLMs and will be crucial to define when the technology is ready for clinical implementation. Educating health care professionals on generative AI will be needed to guarantee responsible integration of the technology, ultimately benefiting patients. LLMs have shown rapid advancements in recent years and are expected to continue improving. Future studies also need to investigate whether their application leads to an improvement in outcome.

## Appendix

Multimedia Appendix 1 Technical details for running the large language model.

Multimedia Appendix 2 Additional figures.

## Abbreviations

AI: artificial intelligence
DEGRO: German Society for Radiation Oncology
ISROI: International Society for Radiation Oncology Informatics
LLM: large language model
USMLE: United States Medical Licensing Examination

## Data Availability

The datasets generated during this study are available in the GitHub repository.
