# Supplementary material for: Comparative Evaluation of a Medical Large Language Model in Answering Real-World Radiation Oncology Questions: Multicenter Observational Study
Source: J Med Internet Res. 2025 Sep 23;27:e69752. doi: 10.2196/69752 (PMC12504895; doi:10.2196/69752)
Supplement: Multimedia Appendix 1 [file jmir_v27i1e69752_app1.docx]

Appendix 1: Technical details for running the LLM

The model OpenBioLLM-70B is a medically fine-tuned Llama3 model. Details on the model have been published online (1).

The original model is over 100GB large and requires considerable hardware resources. To run the model with limited resources on a local system a quantized 5-bit GGUF version of the model was used (2). The open-source tool llama.cpp was used to run the model (3). A simple script was developed and run on a Mac Studio with an Apple M2 Max. The python script is available at *https://github.com/med-data-tools/LLM-evaluation-in-RO*.

The output of an LLM is dependent on the provided prompt (=text input) given to the model. The same prompt (except for the question itself) was used for all 50 questions used in the study. No systematic optimization of the prompt was done and the final prompt was selected after a few initial unsystematic tries. Basic instruction for the LLM were to give a factual correct, helpful and brief answer.

The used prompt was

"<|begin_of_text|><|start_header_id|>system<|end_header_id|>

You are a radiation oncology specialist. You are asked a question by a colleague. You give a factual correct, helpful and concise answer. The answer should be very brief.<|eot_id|><|start_header_id|>user<|end_header_id|>**QUESTION**<|eot_id|><|start_header_id|>assistant<|end_header_id|>The answer is:”

**QUESTION** was replaced by the individual of the 50 questions being asked in the study. The structure and the special tokens (e.g.; “<|begin_of_text|>”) were used based on the prompting format of Llama3 as published by Meta AI (4).

The output of the model was limited to 400 tokens, which corresponds to about 300 words. The answers of the model were used as provided by the model and not changed in any way for the evaluation part.

**References**

1. Huggingface: Llama3-OpenBioLLM-70B [Internet]. Available from: https://huggingface.co/aaditya/Llama3-OpenBioLLM-70B

2. Huggingface: Llama3-OpenBioLLM-70B-GGUF [Internet]. Available from: https://huggingface.co/mradermacher/OpenBioLLM-Llama3-70B-GGUF

3. Github: Llama.cpp [Internet]. Available from: https://github.com/ggerganov/llama.cpp

4. Meta AI: Llama3 Model Card [Internet]. Available from: https://www.llama.com/docs/model-cards-and-prompt-formats/meta-llama-3/
