# Supplementary material for: Comparative Evaluation of a Medical Large Language Model in Answering Real-World Radiation Oncology Questions: Multicenter Observational Study
Source: J Med Internet Res. 2025 Sep 23;27:e69752. doi: 10.2196/69752 (PMC12504895; doi:10.2196/69752)
Supplement: Multimedia Appendix 2 [file jmir_v27i1e69752_app2.docx]

Supplementary Figures

Figure S1: Comparison of results (mean quality score) between clinical experts and LLM assessed by questioner reviewer on different domains. The dashed blue lines indicate the results of the best and worst clinical expert.


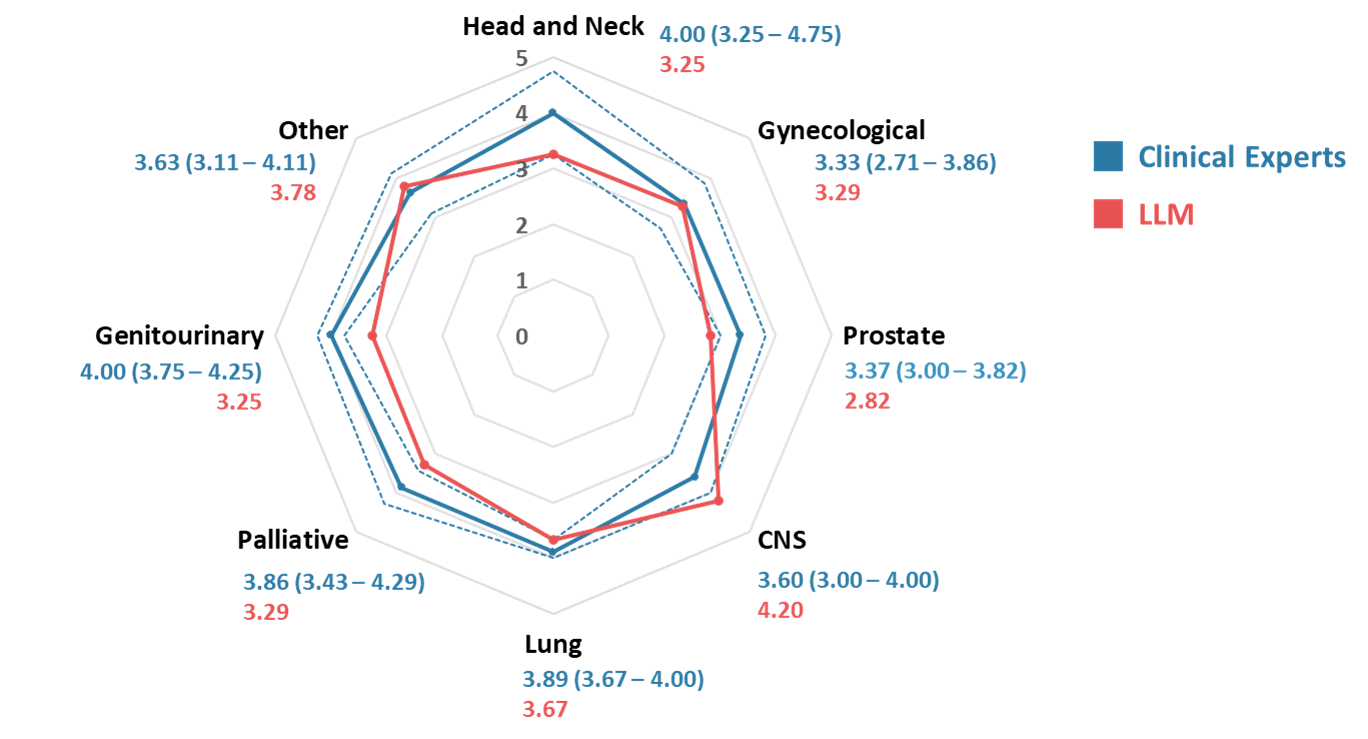


Abbreviations: CNS – central nervous system, LLM: large language model

Figure S2: Comparison of results (mean quality score) between clinical experts and LLM assessed by second reviewer on different domains. The dashed blue lines indicate the results of the best and worst clinical expert.


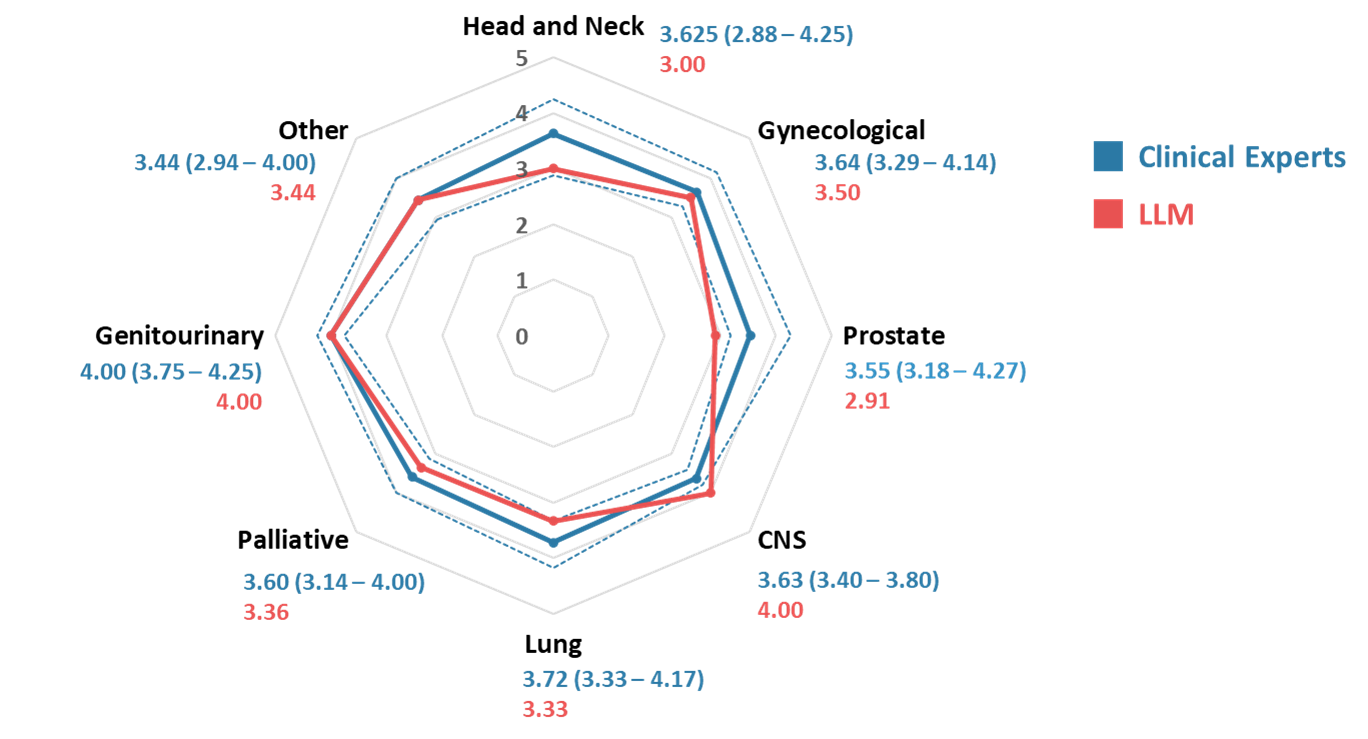


Abbreviations: CNS – central nervous system, LLM: large language model

Figure S3. Quality of the answers as assessed by the second reviewer. A – Box plots with violin plots for comparison of quality score between LLM and mean as well as individual clinical experts. B – Association of the quality of answers with their source and difficulty.


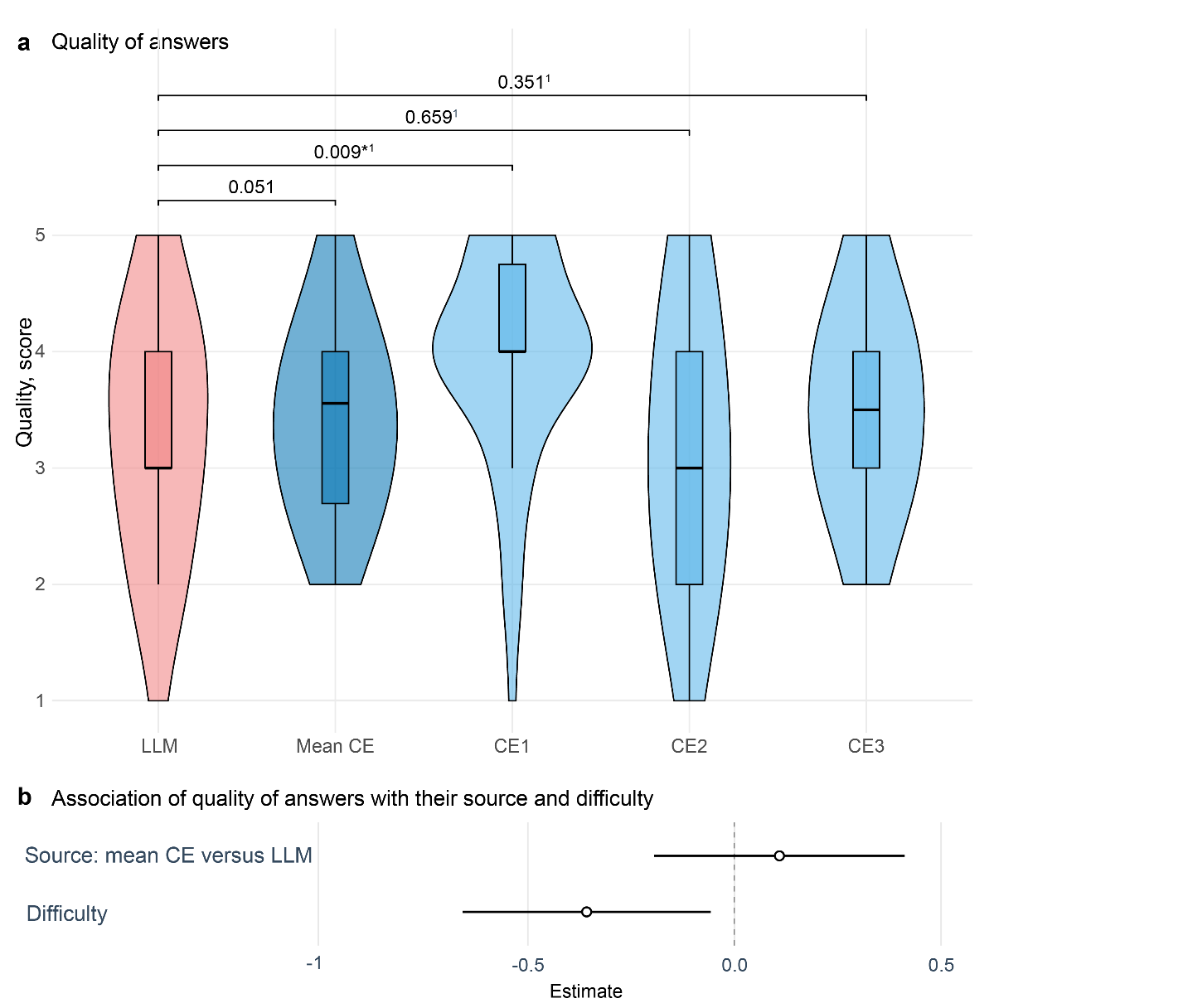


Abbreviations: CE – clinical expert, LLM – large language model. ^1^Wilcoxon signed-rank test corrected for multiple comparisons with a false-discovery rate.

Figure S4. Percentages of answers deemed “potentially harmful” by the second reviewer.
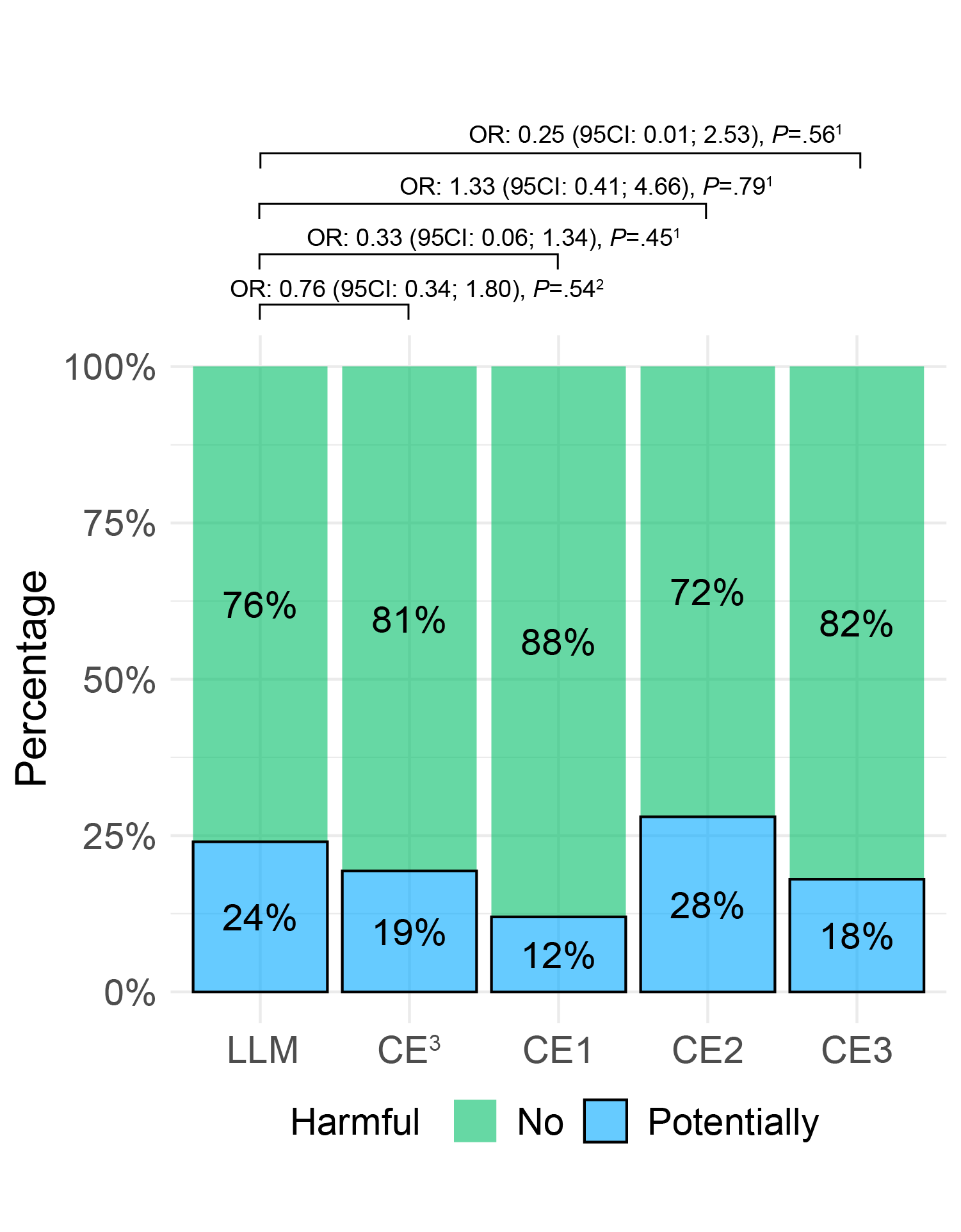


^1^McNemar test corrected for multiple comparisons with a false-discovery rate. ^2^Fisher Exact test. ^3^Cumulative value for clinical experts. Abbreviations: CE – clinical expert, LLM – large language model, OR – Odds ratio

Figure S5. Percentages of correct and incorrect identifications of the source (LLM or clinical expert) by the second reviewer.


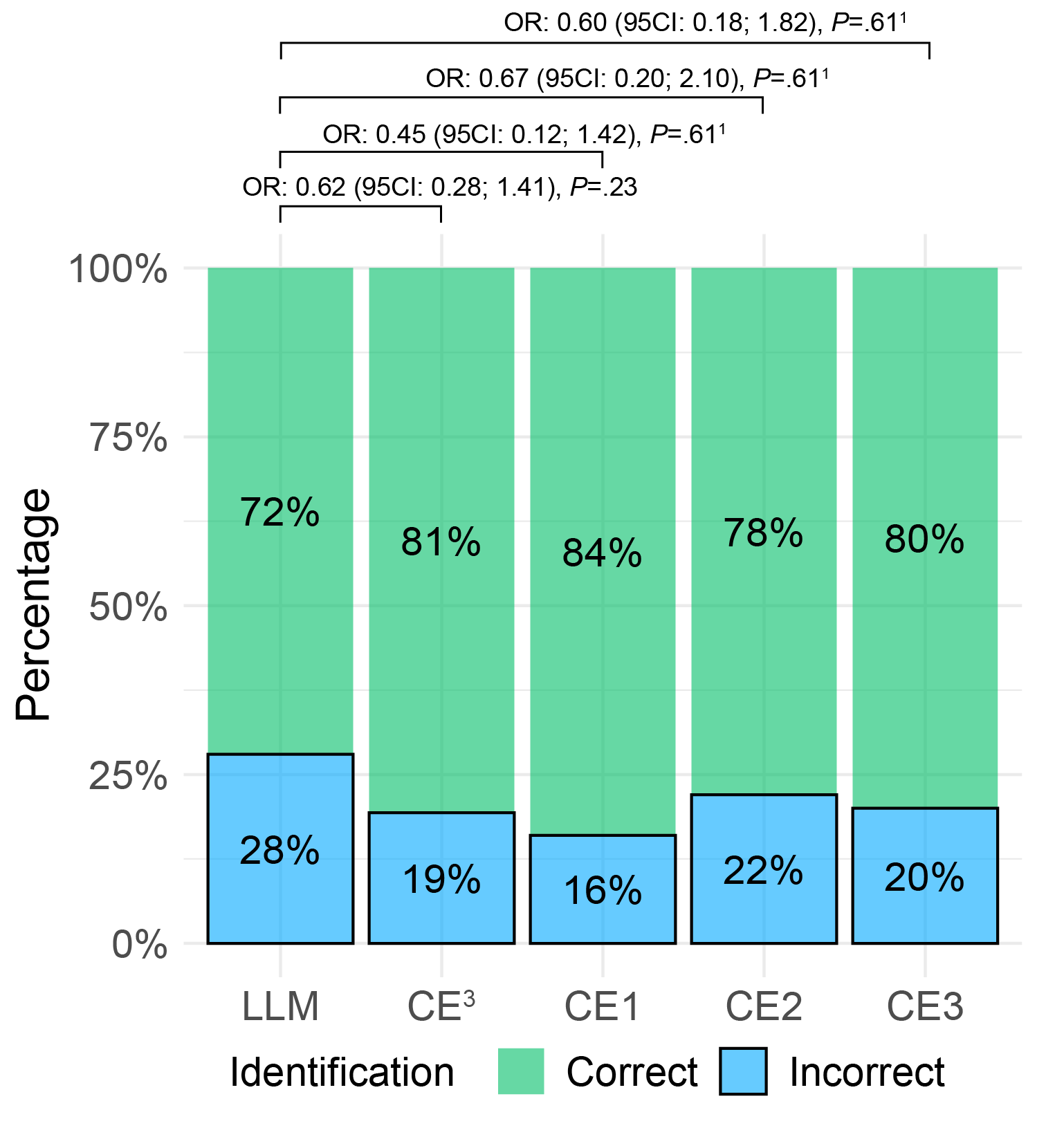


^1^McNemar test corrected for multiple comparisons with a false-discovery rate. ^2^Fisher Exact test. ^3^Cumulative value for clinical experts. Abbreviations: CE – clinical expert, LLM – large language model, OR – Odds ratio
